# Supplementary material for: Factors shaping Covid-19 vaccine acceptability among young people in South Africa and Nigeria: An exploratory qualitative study
Source: PLOS Glob Public Health. 2025 Mar 18;5(3):e0003795. doi: 10.1371/journal.pgph.0003795 (PMC11918360; doi:10.1371/journal.pgph.0003795)
Supplement: S1 Table — (DOCX) [file pgph.0003795.s001.docx]

**S1 Table: Participant characteristics**

| **Characteristics** | **Frequency (163)** |
| --- | --- |
| **Age**  15-19  20-24 | 70  93 |
| **Gender**  Female  Male | 125  38 |
| **Place of residence**  Urban  Rural | 79  84 |
| **Occupation**  Unemployed  Employed  Self employed | 145  11  7 |
