# Supplementary material for: Factors shaping Covid-19 vaccine acceptability among young people in South Africa and Nigeria: An exploratory qualitative study
Source: PLOS Glob Public Health. 2025 Mar 18;5(3):e0003795. doi: 10.1371/journal.pgph.0003795 (PMC11918360; doi:10.1371/journal.pgph.0003795)
Supplement: Attachment — Submitted filename: Response to editors 3 October 2024.docx [file pgph.0003795.s004.docx]

**RESPONSE TO EDITORS**

3^rd^ October 2024

Dear Dr. Casale,

Thank you for submitting your manuscript entitled "Factors shaping Covid-19 vaccine acceptability among young people in South Africa and Nigeria: An exploratory qualitative study" to PLOS Global Public Health. Your manuscript files have been checked in-house, but before we can proceed we need you to address the following issues:

***Authors:*** *Thank you for this opportunity to make these additional small changes and resubmit. Please see responses to specific comments below.*

1. Please amend your detailed online Financial Disclosure statement. This is published with the article. It must therefore be completed in full sentences and contain the exact wording you wish to be published.

a) State the initials, alongside each funding source, of each author to receive each grant (if applicable). For example: "This work was supported by the National Institutes of Health (####### to AM; ###### to CJ) and the National Science Foundation (###### to AM)."
b) State what role the funders took in the study. If the funders had no role in your study, please state: “The funders had no role in study design, data collection and analysis, decision to publish, or preparation of the manuscript.

***Authors:*** *The Financial Disclosure statement has now been updated in line with the indications above.*

2. Please ensure that the funders and grant numbers match between the Financial Disclosure field and the Funding Information tab in your submission form. Note that the funders must be provided in the same order in both places as well.

***Authors:*** *We have double checked this and confirm that the funders are listed in the same order in both places.*

3. Please update your online Competing Interests statement. If you have no competing interests to declare, please state: “The authors have declared that no competing interests exist.”

***Authors:*** *the competing interests statement has been updated as suggested*.

4. Thank you for providing a detailed explanation of the data availability in your study. We understand the sensitivity of the data and the need to protect the privacy of the participants.

As per PLOS policy, we do allow for exceptions in cases where there are ethical or legal considerations with respect to the public sharing of the underlying data (<https://journals.plos.org/globalpublichealth/s/data-availability#loc-human-research-participant-data-and-other-sensitive-data>).

However, to ensure the integrity of the research and to allow for the possibility of data access by other researchers under controlled conditions, we request the authors to consider the following:

a) Please provide contact information for a Data Access Committee, Ethics Committee, or other institutional body to which data requests may be sent from interested researchers. This committee would be responsible for reviewing requests and ensuring they meet relevant guidelines before any data is shared.
b) If the above (a) is not possible, is there any institutional representative(s) who did not collaborate in the study and are not listed as authors on the manuscript, but who would be able to hold the data and/or respond to external requests for data access, please provide their contact information in your data availability statement.

We will update your Data Availability statement on your behalf to reflect the information you provide.

***Authors:*** *We thank the Editors for these suggestions. We prefer to not further burden our Ethics Committee, however have identified an institutional representative, who is not a co-investigator or co-author and is willing to hold the data and respond to requests for access. He is a full Professor at the School of Public Health at the University of the Western Cape and has served for many years on its Biomedical Ethics Committee. His name and details are now included in our updated data availability statement.*


5. Please provide separate figure files in .tif or .eps format only, not as Word document, and ensure that all files are under our size limit of 10MB.

For more information about how to convert your figure files please see our guidelines: <https://journals.plos.org/globalpublichealth/s/figures>

***Authors:*** *These figures in the required format have now been provided*.


6. Main Tables should not be uploaded as individual files. Please remove these files and include the Tables in your manuscript file as editable, cell-based objects. For more information about how to format tables, see our guidelines: <https://journals.plos.org/globalpublichealth/s/tables>

***Authors:*** *The (one) main table, Table 1, has now been included in the manuscript file as suggested.*

7. We have noticed that you have uploaded Supporting Information files ("Table S1 140924.docx"), but you have not included a list of legends. Please add a full list of legends for your Supporting Information files before or after the references list.

***Authors:*** *This has now been included for the (one) supplementary table, just before the reference list.*
