# Supplementary material for: Factors shaping Covid-19 vaccine acceptability among young people in South Africa and Nigeria: An exploratory qualitative study
Source: PLOS Glob Public Health. 2025 Mar 18;5(3):e0003795. doi: 10.1371/journal.pgph.0003795 (PMC11918360; doi:10.1371/journal.pgph.0003795)
Supplement: Attachment — Submitted filename: Review 2.docx [file pgph.0003795.s003.docx]

Dear Authors,

Thank you for your hard work and this end product is a great improvement. Most of my concerns were addressed, however I am still a little concerned with the presentation of your analysis – please see my attached comments.

**Data analysis**

We analysed the data through a collaborative group process. The analysis was conducted with the support of Dedoose software, which allowed team members to work simultaneously online and from different locations. The main analytical approaches employed were thematic analysis and template analysis (50-52). The data were analyzed by means of an iterative process incorporating aspects of both deductive and inductive approaches (53-55). The data analysis was conducted by four of the authors, in consultation with the bilingual interviewers who conducted the focus groups in KwaZulu-Natal. An initial broad analytical framework was first developed, based on the nine components of the acceptability theoretical framework that had also informed the design of the topic guides and interview schedules. Transcripts were divided among the four researchers on the team. All researchers read all the transcripts, without coding, to familiarize themselves with the data and make note of what stood out as interesting. Each researcher then coded three transcripts, of which at least one from a focus group. Initial themes were identified from the transcript content, in terms close to the language used by respondents, and attention was paid to the way the themes were treated and presented (56). The team then met virtually to compare and discuss their initial list of generated codes, and cluster these within overarching themes based on the 9 model components. Themes that did not seem to fit well within this framework were ‘parked’ for further discussion. Potential subthemes were also identified. After an analytical framework was agreed on, this was uploaded as a code tree using Dedoose software, with agreed definitions for each code to guide the coders. The researchers then divided the remaining transcripts to code. Regular virtual meetings were held over a period of two months, to identify, refine and review themes, resolve challenges and iteratively refine the analytical framework. This approach allowed for potential additional themes or ‘components’ to be added, as well as for sub-themes to emerge.
