# Supplementary material for: Factors shaping Covid-19 vaccine acceptability among young people in South Africa and Nigeria: An exploratory qualitative study
Source: PLOS Glob Public Health. 2025 Mar 18;5(3):e0003795. doi: 10.1371/journal.pgph.0003795 (PMC11918360; doi:10.1371/journal.pgph.0003795)
Supplement: Attachment — Submitted filename: Authors response to reviewers 091224.docx [file pgph.0003795.s005.docx]

**Authors’ response to additional review comments**

9 December 2024

**Reviewer #1:** The authors have carefully considered and responded to the comments of the reviewers and the paper is reading well.

***Authors:*** *We thank the reviewer for the positive comments, and for carefully reviewing this revised paper.*

A few very minor points.
A final check for the use of adolescents, youth and young people to ensure consistency. A sentence describing the choice of terms in the methods would be appropriate.

***Authors:*** *We have now indicated, on page 8 under the ‘theoretical framework’ section:*

*“our definition of ‘young people’ for the model development process and this paper refers to individuals between 10 and 24 years of age, comprising the partly overlapping categories of adolescents (10-19) and youth (15-24).”*

*Given these definitions, we use the terms ‘young people’ and ‘adolescents and youth’ interchangeably in this paper, to avoid too much repetition of the same terms. We have also carefully gone through the paper once again to check for the consistent use of terms, and have made changes where appropriate.*

The limitations of the study are very comprehensive and are very helpful. However a line in the methods under data analysis that describes why there is no country comparison or alternatively a reference to the section on limitations will facilitate the reader understanding this earlier in the paper.

***Authors:*** *Thank you for this insightful comment. We have now added the following text at the end of the ‘data analysis’ section, on pages 17 and 18:*

“*While we paid attention to areas of disagreement in the data, the study design, sub-sample sizes and breadth of the analysis, did not allow us to properly explore differences in perceptions across race, gender, countries or (urban versus rural) locations, as explained further in the limitations section (below).”*

Line 423 has 'the' missing in front of the word field.

***Authors:*** *Thank you for picking this up. We have now corrected this sentence.*

The IDI respondents are better educated and have more opportunities. This distinction could be made clearer around line 450.

***Authors:*** *Thank you for this useful suggestion. We have now added the following text, on page 19, in the last paragraph of the section describing participant characteristics:*

*“It is clear from this demographic information that IDI participants were overall older than the FGD participants, better educated and had more work opportunities, in part as a result of their (more urban) geographical location.”*

At line 655 under the theme burden I think it would be helpful to refer back to the period in which the data was collected and the limited coverage of the Nigerian vaccination campaign to provide context to the comments and perhaps a contrast with the South African experience.

***Authors:*** *On going back to the data related to these themes, we noted that these challenges were raised by young people across all sites, but predominantly by young people in Nigeria and rural South Africa. We have included this point when introducing the ‘burden’ base theme in the results section, and have also extended the text in the discussion section (page 42) so that it now reads as follows:*

*“Some young people discussed challenges related to transport to and constraints at vaccine sites. These challenges were raised mainly by participants residing in Nigeria and rural South Africa and should be considered in light of the more limited coverage of the Nigerian vaccination campaign at the time of data collection (early 2022).”*

**Reviewer #2:** Most of my comments have been addressed and the changes have made for a great article. Unfortunately, the presentation of the methods requires further minor work for consistency and clarity. Please see my attached comments.

***Authors:*** *We thank the reviewer for the positive comments. We agree that the review and revisions have indeed made the paper stronger, and are grateful to the reviewers for their time and attention to detail. Regarding the reviewer’s additional very useful comments related to the presentations of the methods, we trust we have adequately addressed these in the revised ‘data analysis’ section (see pages 16-18 of the revised paper with track changes). We realise we were not using terminology precisely and consistently, as indicated by the reviewer, which indeed made this section confusing and difficult for the reader to follow. Following some additional reading on the presentation of qualitative analysis, we decided to use the terms ‘base themes’ to denote the overarching themes derived from the component constructs of our ‘Accelerate framework’, and ‘sub-themes’ to refer to the more specific themes that fit within these overarching themes. We trust we have now defined and explained these more clearly in the description of the data analysis. We have also now referred to ‘template(s)’ versus ‘framework(s)’, and ‘codes’ versus ‘themes’ where appropriate.*
